# Substantial enhancement of *Agrobacterium*-mediated transgene-free genome editing via short-term chemical selection using citrus as a model plant

**DOI:** 10.1093/hr/uhaf153

**Published:** 2025-09-19

**Authors:** Yanjun Li, Zongrang Liu, Frederick G Gmitter Jr., Zhanao Deng, Baoping Cheng, Hui Duan, Yi Li

**Affiliations:** Department of Plant Science and Landscape Architecture, University of Connecticut, 105 Ahern Lane, Storrs, CT 06269, USA; Xianghu Laboratory, Biotechnology Research Institute, No. 3300, Benjing Avenue, Qianwan Bioport Phase I, Xiaoshan District, Hangzhou， Zhejiang 311215, China; Appalachian Fruit Research Station, Agricultural Research Service, U.S. Department of Agriculture, 2217 Wiltshire Road, Kearneysville, WV 25430, USA; Xinjiang Institute of Ecology and Geography, Chinese Academy of Sciences, 818 South Beijing Road, Urumqi, Xinjiang 830011, China(current); Citrus Research and Education Center, University of Florida, 700 Experiment Station Road, Lake Alfred, FL 33850, USA; Department of Environmental Horticulture, Gulf Coast Research and Education Center, IFAS, University of Florida, 14625 County Road 672, Wimauma, FL 33598-6101, USA; Guangdong Provincial Key Laboratory of High Technology for Plant Protection, Plant Protection Research Institute, Guangdong Academy of Agricultural Sciences, 29 Jinying Road, Tianhe District, Guangzhou, Guangdong 510640, China; USDA-ARS, U.S. National Arboretum, Floral and Nursery Plants Research Unit, Beltsville Agricultural Research Center (BARC)-West, 10300 Baltimore Avenue, Beltsville, MD 20705, USA; Department of Plant Science and Landscape Architecture, University of Connecticut, 105 Ahern Lane, Storrs, CT 06269, USA

## Abstract

Citrus production is threatened by biotic and abiotic stresses, particularly Huanglongbing (HLB), creating an urgent need for efficient engineering of citrus for disease resistance. Gene editing, especially transgene-free approaches, offers a promising alternative to traditional breeding, which is slow and constrained by citrus’ long juvenile phase. However, producing transgene-free, genome-edited citrus remains challenging. Here, we present a novel method to significantly enhance the efficiency of transgene-free gene editing in citrus using *Agrobacterium*-mediated transient expression of Cas9 and gRNAs. By treating *Agrobacterium* cells and citrus explants and applying a 3-day transient kanamycin selection, we achieved a 17-fold increase in transgene-free editing efficiency. The transient kanamycin-mediated suppression of shoot regeneration from non-*Agrobacterium*-infected cells not only improved the efficiency of identifying edited plants but also enhanced shoot regeneration efficiency from *Agrobacterium*-infected cells, regardless of whether these cells had stably incorporated T-DNA or not. This enhancement was likely due to reduced competition for space and nutrients from shoots regenerated from noninfected cells. In experiments targeting the phytoene desaturase (*PDS*) gene, transgene-free mutant shoot recovery increased from 0.017% to 0.291% of the total shoots produced. With an efficient screening method for gene-edited plants, the development of transgene-free gene-edited plants becomes relatively easy and practicable. These results suggest that this optimized protocol could be applicable to other perennial crops, offering a valuable tool for improving citrus varieties and other economically important plants.

## Introduction

Citrus is a major tropical and subtropical fruit tree, cultivated in ~140 countries. However, emerging biotic and abiotic stresses, particularly Huanglongbing (HLB), have posed a significant threat to global citrus production [1–4]. This crisis underscores the urgent need for developing novel citrus varieties with traits capable of countering these challenges. While conventional cross-breeding remains a vital approach, this process is too slow and time-consuming, taking decades due to the biological limitations of citrus [5]. Gene editing offers a powerful alternative for improving citrus genetics, including the development of HLB-resistant varieties [6].

In annual crops, transgenes such as Cas9 can be easily eliminated through genetic segregation by the second generation [7]. However, in citrus and other perennial crops with long juvenile phases, producing transgene-free edited plants remains a significant challenge, but it is a priority for both the scientific community and the citrus industry [8, 9]. Although successful methods for generating transgene-free, genome-edited citrus and other plants have been reported [10–19], alternative methods and more efficient transgene-free editing processes are crucial. This need is particularly urgent given the devastating impact of HLB, which has destroyed >70% of citrus trees in Florida, a major citrus-producing region both in the USA and globally [1, 20].

We previously developed a method using *Agrobacterium*-mediated transient expression of Cas9 and gRNA to produce transgene-free, genome-edited plants in tobacco [21]. Since then, numerous labs have successfully applied this technique to produce nontransgenic, gene-edited plants of various species [22–26]. However, when we applied this method to citrus, we observed low efficiencies in generating transgene-free, genome-edited plants (unpublished data).

More recently, we demonstrated that pretreating *Agrobacterium* cells and citrus explants significantly enhanced both stable transgenic citrus plant production and the transient expression of a GUS reporter gene [27, 28]. Building on these findings, we applied the same treatments to improve the transient expression of Cas9 and gRNA, aiming to enhance the efficiency of transgene-free gene editing in citrus. To further optimize the screening process, we introduced a transient kanamycin treatment to suppress shoot regeneration from cells that were not infected by *Agrobacterium*. Compared to our previous method [21], this combined approach resulted in a 17-fold increase in the efficiency of transgene-free gene editing, yielding one transgene-free gene-edited shoot per epicotyl explant and 0.3% of all shoots produced. Here, we present the enhanced method and compare its efficiency in producing transgene-free, genome-edited citrus shoots with that of standard treatments lacking these improvements.

**Figure 1:**
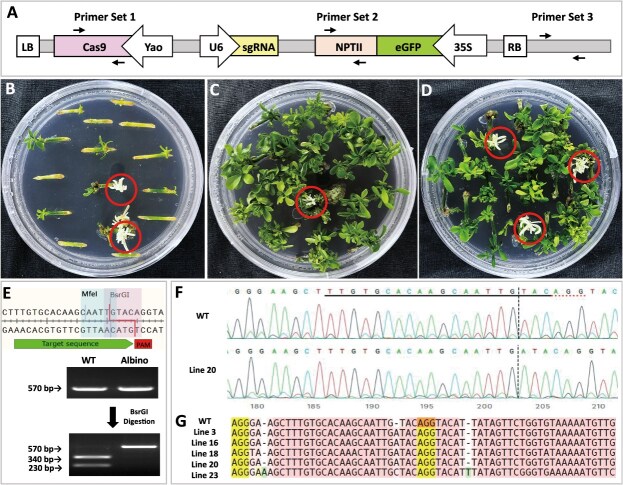
Regeneration of *pds* mutant shoots from the *Agrobacterium*-infected citrus epicotyl explants with or without transient kanamycin selection. (**A**) Schematic illustration of the binary gene editing construct. The gene construct is composed of three transcriptional units: *Yao::Cas9*, *U6::sgRNA* targeting the *PDS* gene and the *35S::eGFP-NPTII* fusion. The detailed information is described previously [29]. Three sets of primer pairs and their positions in vector are indicated. (**B–D**) Albino shoots regenerated under constant selection with 100 mg/l kanamycin (**B**), no kanamycin selection pressure (**C**), and a 3-day transient kanamycin selection (**D**). (**E**) The sgRNA sequence and a restriction site of the BsrGI enzyme in the gRNA region near PAM sequence.

## Results

### Enhanced the recovery of *PDS* mutants via transient kanamycin selection

In this study, we targeted the phytoene desaturase (*PDS*) gene for editing due to its ease of identification and its common use in the development of gene-editing protocols for higher plants. The *NPTII* gene (Fig. 1A), incorporated in the Ti-plasmid alongside *Cas9* and gRNAs, confers kanamycin resistance. Transient kanamycin resistance is expected in the early stages following *Agrobacterium* infection, as the NPTII gene should be transiently expressed [21, 28]. In contrast, noninfected cells remain sensitive to kanamycin, allowing selective inhibition of their division and growth within a reasonable time frame.

In our experiment, we applied 100 mg/l kanamycin either temporarily (for 3 days) or continuously, following a 3-day co-incubation with *Agrobacterium*. When kanamycin was continuously used as a selection agent during the shoot regeneration process, a high proportion of the shoots were albino (putative *pds* mutants), while the number of green shoots was very low (Fig. 1B). In the absence of kanamycin, a large number of shoots were regenerated, with only a minimal occurrence of albino shoots (Fig. 1C). When kanamycin was applied for 3 days, we observed a significant increase in albino shoots and reductions in the total number of regenerated shoots (Fig. 1D). The editing/mutations of the *PDS* gene in these albino shoots were confirmed by DNA digestion and Sanger sequencing analysis (Fig. 1E–G).

As shown in Table 1, without kanamycin selection, 0.859% of shoots were *PDS* mutants on a per explant basis, compared to 2.89% with 3 days of transient kanamycin selection, representing an ~300% increase in *PDS* mutant shoot production. One explanation is that the 3-day kanamycin treatment reduced shoot production from non-*Agrobacterium*-infected cells, allowing *PDS* mutant cells to face less competition for shoot regeneration.

**Table 1:** Effects of various treatments on transgene-free editing efficiency

Treatment^a^	Total *pds* shoots /explant (%)	Transgene-free *pds* shoots /explant (%)	Transgene-free *pds* shoots/total shoots (%)
Control /no Kan^b^	0.859 ± 0.076	0.131 ± 0.030	0.017 ± 0.006
Control/3-D Kan^b^^,^^c^	2.839 ± 0.503	0.387 ± 0.140	0.087 ± 0.028
Pretreatment + LA (10 uM) + 3-D Kan^d^	7.899 ± 1.311	0.708 ± 0.114	0.172 ± 0.029
Pretreatment + PBZ (30 uM) + 3-D Kan^d^	9.031 ± 2.212	0.963 ± 0.453	0.229 ± 0.093
Pretreatment + SMZ (30 uM) + 3-D Kan^d^	8.242 ± 1.609	1.028 ± 0.364	0.244 ± 0.073
Pretreatment + SMZ, LA, and PBZ + 3-D Kan^d^	10.463 ± 2.888	1.182 ± 0.387	0.291 ± 0.100

a All experiments had three to four replicates. For the control experiment, each replicate had 5000–6000 explants. For the rest of the treatments, each replicate had 500–700 explants.

b For the control transformation, citrus explant from etiolated seedlings were cut and used for *Agrobacterium* infection immediately; explants were cultured for 3 days on the solid cocultivation medium before being transferred to the citrus SRM without kanamycin.

c 3-D Kan: 3-day kanamycin treatment (100 mg/l) immediately after 3 days’ coculture with *Agrobacterium* cells, followed with shoot regeneration without kanamycin.

d Pretreatment: Citrus explants were pretreated with a liquid MS medium for 3 h prior to co-incubation with *Agrobacterium* cells. Meanwhile, *Agrobacterium* cells were conditioned in a 1/10 MS liquid medium with 20 mg/l AS for 6 h at 25°C before infection.

### Enhancing transgene-free gene editing in citrus through optimized treatments and transient kanamycin selection

In our previous studies, we optimized several factors to enhance transient expression during *Agrobacterium* infection, identifying treatments that significantly increased stable transgenic plant production [27] and transient expression of a reporter gene hosted in the T-DNA region [28]. In this study, we introduced a 3-day transient selection and combined it with optimized treatments to enhance the efficiency of *Agrobacterium*-mediated transgene-free editing. Etiolated epicotyls from 4-week-old seedlings were cut into 1-cm segments and incubated for 3 h in liquid medium containing 1 mg/l 2,4-Dichlorophenoxyacetic acid (2,4-D), 3 mg/l 6-benzylaminopurine (BAP), and 0.1 mg/l 1-Naphthaleneacetic acid (NAA). *Agrobacterium* cells were pretreated in 1/10 MS liquid medium (pH 5.6) with 20 mg/l acetosyringone (AS) for 6 h prior to cocultivation. To further enhance gene expression, 30 μM sulfamethazine (SMZ), 30 μM paclobutrazol (PBZ), and 10 μM lipoic acid (LA) were added to the co-cultivation medium (CCM).

As shown in Table 1, the treatments and their combinations, along with the 3-day kanamycin treatment, significantly increased both total gene editing and transgene-free editing efficiencies compared to their relevant controls. Specifically, for transgene-free gene editing, these combinations resulted in 1.182% transgene-free *PDS*-edited plants based on the number of explants used, and 0.291% transgene-free *PDS*-edited shoots based on the number of shoots produced, compared to 0.131% and 0.017% in the controls, respectively. This represents 9- and 17-fold increases over the controls without these treatments.

### Efficient identification of edited and transgene-free edited plants

#### Identification of edited plants

Our initial method for identifying gene-edited shoots involved BsrGI restriction enzyme digestion, targeting the BsrGI recognition site located just upstream of the PAM sequence (Fig. 1A). We utilized polymerase chain reaction (PCR) amplification followed by BsrGI digestion to detect mutations (Fig. 1E). Undigested PCR fragments indicate that the *PDS* gene was successfully edited, while digested PCR products suggest the *PDS* gene remained unedited. In wild-type shoots, the PCR product was digested into two fragments of 340 and 230 bp, whereas undigested PCR fragments in edited shoots remain at 570 bp (Fig. S1).

#### Identification of transgene-free edited plants

**Figure 2:**
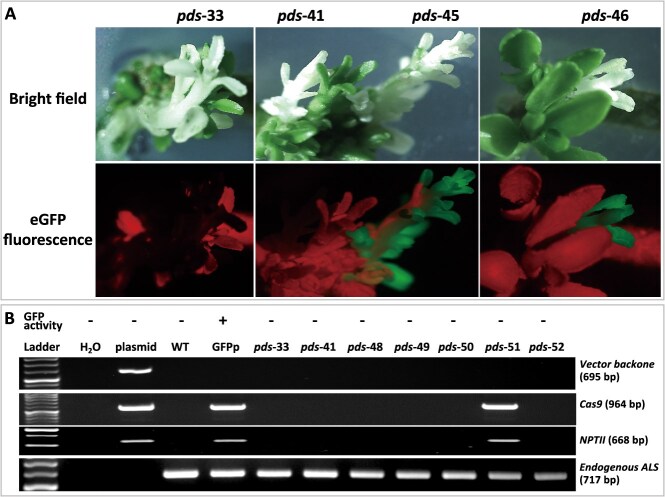
Verification of transgene absence in *pds* mutant shoots via eGFP fluorescence and PCR analysis. (**A**) Selected *pds* mutant shoots were initially screened under bright field illumination (top panel) and fluorescence excitation light (bottom panel) to identify putative transgene-free shoots, indicated by the absence of eGFP fluorescence. (**B**) Shoots that were eGFP-negative were further analyzed by PCR to confirm the absence of transgenes. Four primer pairs were used, targeting the *Cas9* and *NPTII* genes within the T-DNA region, the vector backbone, and the endogenous citrus *ALS* gene. The presence or absence of eGFP signal and the corresponding mutant code are noted above each lane, while the target genes for PCR amplification are listed on the right side of the image.

To identify nontransgenic, gene-edited mutant plants, we used a combination of reporter gene screening and PCR analysis. The *eGFP* gene, driven by the 35S promoter, allowed for easy and quick visual identification of *eGFP*-positive and *eGFP*-negative shoots. Shoots lacking eGFP fluorescence were then subjected to PCR analysis to confirm the absence of transgene integration. In this study, we analyzed 10 eGFP-negative and 5 eGFP-positive shoots. Our results demonstrate a strong correlation between eGFP fluorescence and PCR-based transgene detection. Representative PCR results for both eGFP-negative and eGFP-positive shoots are shown in Fig. 2B.

In summary, as shown in Table 1, the controls without kanamycin selection showed a very low transgene-free editing efficiency (0.017%). A 3-day kanamycin selection significantly increased this efficiency to 0.087%. Pretreatments of *Agrobacterium* cells and explant tissues, along with chemical treatments after 3 days of co-incubation, further enhanced the efficiency, with SMZ being the most effective individual treatment (0.244%). Combining a 3-day kanamycin selection, pretreatment of *Agrobacterium* cells and citrus explants, and treatment of explant tissues with SMZ, LA, and PBZ after *Agrobacterium*-mediated infection resulted in the highest transgene-free editing efficiency (0.291%) based on the total number of shoots produced.

## Discussion

Efficient generation of transgene-free, gene-edited plants is much needed for citrus and other asexually propagated, heterozygous perennial crops [5, 30]. In this work, we report the development of an effective strategy to significantly improve the efficiency of *Agrobacterium*-mediated transgene-free editing in citrus. This improvement is achieved through pretreatment of both *Agrobacterium* cells and explants before co-incubation, treating explants with various chemicals, and applying a 3-day transient antibiotic selection after 3 days of infection. We have demonstrated an average production efficiency of one transgene-free, gene-edited shoot per epicotyl explant, corresponding to approximately three transgene-free edited shoots out of 1000 regenerated shoots (0.291%).

Although the editing efficiency (0.291% of shoots producing edited plants) may appear low, it represents a 17-fold improvement over the previously published and widely used method by Chen *et al*. [21] and is highly significant given the challenges associated with generating nontransgenic, gene-edited woody perennials. Equally important, the identification of edited plants from a pool of regenerants is straightforward, as demonstrated here using GFP-negative selection combined with restriction enzyme digestion. A variety of efficient and scalable methods has been developed to detect genome edits across mutation types (indels, SNPs, deletions) and screening scales (from small-scale validation to high-throughput analysis). These include T7 endonuclease I (T7EI) and Surveyor assays for mismatch detection in PCR-amplified heteroduplexes [31, 32], high-resolution melting (HRM) analysis for detecting differences in DNA melting curves [33–35], and restriction fragment length polymorphism (RFLP) assays when edits alter restriction sites [36]. Additional tools such as amplicon sequencing (next-generation sequencing) [37, 38], droplet digital PCR (ddPCR) [39, 40], fluorescent PCR-capillary electrophoresis [41], ligation detection reaction (LDR) [42], and polyacrylamide gel electrophoresis (PAGE) [43, 44] are also widely used for the reliable identification of genome-edited plants [45]. Taken together with the widespread adoption of *Agrobacterium*-mediated transformation in citrus due to its simplicity and ease of use, we believe this method holds significant potential for application in citrus. More importantly, the method presented here should be applicable to other woody plant species for efficiently generating transgene-free, gene-edited plants without the need for sexual reproduction.

The improvement of transient gene expression activities [28] and gene editing efficiencies (as reported here) through our treatments of *Agrobacterium* cells and explants, both prior to and after co-incubation, may involve several mechanisms. *Agrobacterium* infection and subsequent transient T-DNA gene expression are known to be influenced by plant hormones and secondary metabolites produced by host plant cells [46], as well as by free radicals generated through explant wounding, *Agrobacterium* infection, and other manipulations [47, 48]. Furthermore, rapid methylation of T-DNAs upon entering the nucleus can negatively regulate the expression of genes within the T-DNA region [47, 49]. Conceptually, combinations of these factors could hinder transient Cas9 and gRNA expression, reducing the efficiency of transgene-free gene editing. Consequently, our pretreatments of *Agrobacterium* cells and citrus explants, along with chemical treatments of explants following *Agrobacterium* infection, have positively influenced transient gene expression in the T-DNA region [28], significantly enhancing the overall effectiveness of transgene-free gene editing.

It has been reported that the addition of a DNA methylation inhibitor, azacitidine (5-AzaC), can enhance transient gene expression [47]. Our previous study also demonstrated that SMZ, another methylation inhibitor, significantly increased transient GUS expression in both epicotyl and stem tissues of citrus [28]. We hypothesize that SMZ may inhibit rapid methylation of T-DNAs, thereby promoting transient expression of genes located within the T-DNA region. The enhancement of transient expression by LA in citrus may be attributed to its antioxidant properties. Previous studies have shown that *Agrobacterium* infection can induce cell death and tissue browning, which negatively impact transformation efficiency [50]. As a known antioxidant, LA has been used to improve stable transformation in various species, including soybean, tomato, wheat, and cotton [51–53]. It is therefore plausible that LA reduces excessive reactive oxygen species (ROS) accumulation in *Agrobacterium*-infected plant cells, thereby enhancing transient gene expression. However, the precise mechanism remains unclear. Similarly, PBZ is recognized as a multistress ameliorant [54, 55], offering protection against both abiotic and biotic stresses, including those induced by *Agrobacterium* infection. In addition, PBZ functions as a plant growth regulator [56] and has been reported to promote callus formation and somatic embryogenesis [57, 58]. We propose that the combined stress-protective and growth-regulating effects of PBZ, whether applied alone or in combination, enhance transient gene expression and support regeneration of transformed citrus cells.

We have demonstrated that a 3-day transient kanamycin selection pressure enhances the production efficiency of transgene-free gene editing. A possible explanation for this is that transient kanamycin selection suppresses the shoot development from non-*Agrobacterium*-infected cells, thereby reducing the competition between non-gene-edited and gene-edited shoots. While a 3-day treatment appears optimal for producing transgene-free, gene-edited citrus in our study, different plant species or citrus varieties may require shorter or longer selection periods. Additionally, alternative selection agents, such as hygromycin or herbicides, may necessitate varying selection durations, which would require further testing. We believe that the introduction of transient selection pressure represents a significant step in improving the efficiency of both the production and identification of transgene-free, gene-edited citrus and other plant species.

Transgene-free, gene-edited citrus plants have been achieved using the Cas12a/crRNA RNP complex delivered into embryogenic protoplasts [13, 14]. However, this method involves complex, inefficient, and difficult procedures for shoot regeneration from protoplasts. More recently, an innovative approach using an *Agrobacterium*-mediated Cas12a/CBE expression system has been employed to co-edit target genes along with the *ALS* gene, which encodes acetolactate synthase [8, 13]. Successful base editing of the *ALS* gene at a specific location can lead to chlorsulfuron resistance, enabling the selection of gene-edited citrus without the need for kanamycin or other selection markers. Since no transgenic plant selection is required, the method relies on the transient expression of Cas12a and gRNAs. However, as a base editor needs to be used, co-editing efficiencies can be very low [13, 59, 60]. We hypothesize that combining our method with co-editing of a herbicide resistance gene could significantly enhance the efficiency of transgene-free gene editing. Testing this hypothesis in citrus and other plant species would be an exciting next step.

In conclusion, we have developed a significantly improved method that employs *Agrobacterium*-mediated transient expression of Cas9 and sgRNA, enabling reliable and efficient production of transgene-free mutants in citrus epicotyl explants. Compared to the widely used method developed by Chen *et al*. [21], our improved method offers a more effective and streamlined protocol. This method has the potential to greatly accelerate the development of transgene-free, genome-edited citrus varieties, particularly for engineering resistance to HLB. Furthermore, it may be broadly applicable to other economically important crops, especially heterozygous perennial species that are difficult to regenerate from protoplasts or through biolistic transformation.

## Materials and methods

### Preparation of plant materials

Seeds of Carrizo citrange [*Citrus sinensis* (L.) Osbeck × *Poncirus trifoliata* (L.) Raf.] were obtained from Tree Source Citrus Nursery (Exeter, CA, USA). Outer seed coats were manually removed, and seeds were surface-sterilized with 75% ethanol for 60 s, followed by 1% sodium hypochlorite for 20 min, then rinsed five times with sterile distilled water. Under sterile conditions, inner seed coats were also removed. The decoated seeds were cultured *in vitro* on MS medium supplemented with 30 g/l sucrose and 7 g/l agar (pH 5.7) at 28°C in the dark for 4 weeks, unless stated otherwise.

### *Agrobacterium* preparation

*Agrobacterium tumefaciens* strain EHA105 carrying the binary vector pYAO::*hSpCas9*_AtU6:*sgRNA(Cs*PDS*)*_CaMV 35S:*e*GFP*-NPTII* (kindly provided by Dr Yannick Jacob, Yale University) was used for transformation. Bacterial stocks were streaked on LB agar plates with 50 mg/l rifampicin and 100 mg/l kanamycin and incubated at 28°C for 2 days. A single colony was inoculated into 2 ml of LB liquid medium with the same antibiotics and shaken at 200 rpm at 28°C for 24 h. This preculture (2 ml) was transferred into 50 ml of fresh LB medium with antibiotics and cultured to an OD_600_ of ~0.6. Bacterial cells were harvested by centrifugation at 5000 rpm for 15 min at 25°C and resuspended in liquid MS medium supplemented with 30 g/l sucrose, 3 mg/l 6-BA, and 20 mg/l AS, unless otherwise specified.

### Pretreatments

Pretreatments of etiolated seedlings and *Agrobacterium* cells have previously been reported [28]. One-centimeter-long epicotyl segments from 4-week-old etiolated seedlings were incubated in a liquid hormone-rich medium containing MS, 1 mg/l 2,4-D, 0.1 mg/l NAA, 3 mg/l 6-BA, and pH 5.7 for 3 h at 25°C before *Agrobacterium* infection. *Agrobacterium* cells were pretreated in a medium with 1/10 MS, 0.5 g/l MES (2-(N-morpholino) ethanesulfonic acid), 20 mg/l AS, and pH 5.6 at 25°C for 6 h prior to infection.

### *Agrobacterium-*mediated transformation and shoot regeneration

The *Agrobacterium*-mediated transformation procedure followed a previously described method [27]. On the day of infection, citrus internodal stems were cut into 1-cm segments under aseptic conditions and immersed in the *Agrobacterium* suspension at 25°C for 10 min. Explants were then blotted dry on sterile filter paper and placed horizontally on CCM containing MS, 3 mg/l 6-BA, 20 mg/l AS, and 30 g/l sucrose, unless otherwise noted. Co-cultivation was carried out in the dark at 25°C for 3 days. Explants were then transferred to shoot regeneration medium (SRM) composed of MS, 3 mg/l 6-BA, 30 g/l sucrose, 150 mg/l Timentin, and 8 g/l agar, unless stated otherwise. Cultures were maintained under a 16-h photoperiod (60 μmol m^−2^ s^−1^) at 26 ± 2°C and subcultured onto fresh SRM every 3 weeks.

To test temporary kanamycin treatment on transgene-free mutant efficiency, after 3 days co-cultivation, explants were transferred to SRM with 100 mg/l kanamycin and cultured under light conditions (60 μmol /m^2^/s) at a 16-h photoperiod (26 ± 2°C) for 3 days. LA (10 μM), PBZ (30 μM), and SMZ (30 μM), individually or in combination, were added to the cocultivation media.

### Using the eGFP reporter system to fast screen transgene-free *pds* mutant plants

The regenerated white shoots (*pds* mutant shoots) were visualized under a fluorescence microscope with 488/525 nm with an excitation/emission filter set (ECLIPSE, TE2000-S, Nikon).

### Molecular confirmation of transgene-free mutant shoots

Genomic DNA was extracted from e*GFP*-negative *pds* mutant shoots. To confirm the absence of T-DNA integration, the following primers were used: Primers 1F (5′-GCCATTCTTCTTCTCGCC-3′) and 1R (5′-AACAAAATCGTTTTCCAGCTTC-3′) to amplify a 964-bp region of the T-DNA fragment. Primers 2F (5′-CGAATCCGCAAAGAATCCC-3′) and 2R (5′-AGGCGGTAGAGAAAACGG-3′) to amplify a 695-bp region of the plasmid backbone. Primers 3F (5′-ATACCGAAAGGTTGGGCAGG-3′) and 3R (5′-TCACCACGATGCCATGTTCA-3′) to amplify a 717-bp fragment of the citrus *ALS* gene. PCR reactions were carried out in 20-μl volumes containing PCR buffer with 1.5 mM MgCl₂, 0.25 mM dNTPs, 0.25 μM of each primer, 0.2 μL e2TAK DNA polymerase (Takara, Japan), and 500 ng of genomic DNA. The thermal cycling conditions were: initial denaturation at 98°C for 5 min; 35 cycles of 98°C for 10 s, primer-specific annealing temperature for 5 s, and 72°C extension; followed by a final extension at 72°C for 10 min. PCR products were visualized on 2% (w/v) agarose gels. The absence of PCR amplification with both primer sets 1 and 2 indicated the lack of T-DNA and vector backbone sequences, confirming that the edited citrus shoots were transgene-free.

### Data analysis

The transgene-free *pds* mutant efficiency is defined as both eGFP-negative and PCR-negative *pds* mutant shoots per total shoots × 100% unless stated otherwise. SPSS software was used for statistical analysis. The statistical significance of the experiments was determined either by the two-tailed Student’s *t*-test with *P* ≤ .05 (comparison between two treatments) or by the single-factor ANOVA with *P* ≤ .05 (comparison among three or more treatments).

## Supplementary Material

Web_Material_uhaf153

## Data Availability

All data included in the main text.
